# Construction and Analysis of an Integrated Regulatory Network Derived from High-Throughput Sequencing Data

**DOI:** 10.1371/journal.pcbi.1002190

**Published:** 2011-11-17

**Authors:** Chao Cheng, Koon-Kiu Yan, Woochang Hwang, Jiang Qian, Nitin Bhardwaj, Joel Rozowsky, Zhi John Lu, Wei Niu, Pedro Alves, Masaomi Kato, Michael Snyder, Mark Gerstein

**Affiliations:** 1 Department of Molecular Biophysics and Biochemistry, Yale University, New Haven, Connecticut, United States of America; 2 Program in Computational Biology and Bioinformatics, Yale University, New Haven, Connecticut, United States of America; 3 Wilmer Institute, Johns Hopkins University School of Medicine, Baltimore, Maryland, United States of America; 4 Department of Genetics, Yale University, New Haven, Connecticut, United States of America; 5 Department of Molecular Cellular Developmental Biology, Yale University, New Haven, Connecticut, United States of America; 6 Department of Genetics, Stanford University, Stanford, California, United States of America; 7 Department of Computer Science, Yale University, New Haven, Connecticut, United States of America; Institute for Systems Biology, United States of America

## Abstract

We present a network framework for analyzing multi-level regulation in higher eukaryotes based on systematic integration of various high-throughput datasets. The network, namely the integrated regulatory network, consists of three major types of regulation: TF→gene, TF→miRNA and miRNA→gene. We identified the target genes and target miRNAs for a set of TFs based on the ChIP-Seq binding profiles, the predicted targets of miRNAs using annotated 3′UTR sequences and conservation information. Making use of the system-wide RNA-Seq profiles, we classified transcription factors into positive and negative regulators and assigned a sign for each regulatory interaction. Other types of edges such as protein-protein interactions and potential intra-regulations between miRNAs based on the embedding of miRNAs in their host genes were further incorporated. We examined the topological structures of the network, including its hierarchical organization and motif enrichment. We found that transcription factors downstream of the hierarchy distinguish themselves by expressing more uniformly at various tissues, have more interacting partners, and are more likely to be essential. We found an over-representation of notable network motifs, including a FFL in which a miRNA cost-effectively shuts down a transcription factor and its target. We used data of *C. elegans* from the modENCODE project as a primary model to illustrate our framework, but further verified the results using other two data sets. As more and more genome-wide ChIP-Seq and RNA-Seq data becomes available in the near future, our methods of data integration have various potential applications.

## Author Summary

The precise control of gene expression lies at the heart of many biological processes. In eukaryotes, the regulation is performed at multiple levels, mediated by different regulators such as transcription factors and miRNAs, each distinguished by different spatial and temporal characteristics. These regulators are further integrated to form a complex regulatory network responsible for the orchestration. The construction and analysis of such networks is essential for understanding the general design principles. Recent advances in high-throughput techniques like ChIP-Seq and RNA-Seq provide an opportunity by offering a huge amount of binding and expression data. We present a general framework to combine these types of data into an integrated network and perform various topological analyses, including its hierarchical organization and motif enrichment. We find that the integrated network possesses an intrinsic hierarchical organization and is enriched in several network motifs that include both transcription factors and miRNAs. We further demonstrate that the framework can be easily applied to other species like human and mouse. As more and more genome-wide ChIP-Seq and RNA-Seq data are going to be generated in the near future, our methods of data integration have various potential applications.

## Introduction

Eukaryotic gene regulation is performed at multiple levels, each distinguished by different spatial and temporal characteristics. The combination and orchestration between regulatory mechanisms in various levels are central to a precise gene expression pattern, which is essential to many critical biological processes [1], [2]. Transcriptional regulation and post-transcriptional regulation, mediated by regulators including transcription factors (TFs) and small non-coding RNAs, such as microRNAs (miRNAs), are two of the most important regulatory mechanisms [3], [4]. At the transcriptional level, TFs bind to promoters and enhancers to either activate or repress gene transcription [4]. At the post-transcriptional level, miRNAs repress the expression of genes by degrading or inhibiting the translation of their target mRNAs [5], [6]. In spite of the dramatic differences in their molecular types, TFs and miRNAs share a common “logic” for the control of gene expression [7]. Both of them are trans-acting factors that function through recognizing and binding specific cis-regulatory elements in DNA or RNA. TFs bind to DNA elements often located in or near their target genes, while miRNAs hybridize to RNA elements mostly located in the 3′ untranslated region (3′UTR) of their target mRNAs. TFs and miRNAs tightly coordinate with each other to ensure accurate and precise gene expression. Furthermore, translated proteins form complexes via physical interactions. These complexes can function only if their constituents are properly regulated. Therefore, each TF or miRNA regulates a large number of interacting target genes [8]–[11] and different TFs and miRNAs control one gene in a combinatorial manner [3], [12], [13]. This essentially forms an integrated gene regulatory network by connecting TFs and miRNAs with their interacting targets. A deep investigation of this network would help to further understand the “language” of gene expression regulation at multiple levels.

Network analysis has proven to be useful in unraveling the complexity of biological regulation [14]–[16]. Different approaches can be employed to gain more insight into the design principles of biological networks. Recently, studies have shown that transcriptional regulation follows a hierarchical organization and regulators at different levels have their own characteristics [17]. In particular, the rearrangement of networks into hierarchy facilitates comparison with other commonplace systems, and provide more intuitive understanding of biological networks [18]. Apart from a top-down approach, one could also study networks via a bottom-up approach by identifying their simple building blocks [19], [20]. These blocks, referred to as network motifs, are patterns that recur within a network at numbers that are significantly higher than expected at random. Examples such as feedback loops and feed-forward loops in transcriptional regulatory networks are found to be conserved in diverse organisms from bacteria to human [20]–[22], and experimentally verified to perform distinct functions like pulse generator and response accelerator [23]. Even though numerous efforts have been placed on the network analysis of biological regulation, most of the earlier studies focused on the transcriptional level. More recently, several system-wide studies have attempted to integrate regulation by TFs at the transcriptional level and that by miRNAs at the post-transcriptional level [24]–[26]. Despite the new insights they provided, the datasets were limited by their coverage and were mostly based merely on computational predictions, which have high false positive rate and were potentially biased.

To overcome the limitations of previous studies, we have used the genome-wide experimental datasets for TF binding created by the model organism encyclopedia of DNA elements (modENCODE) project. The modENCODE project, launched in 2007, aims to generate a comprehensive annotation of functional elements in the *C. elegans* and *D. melanogaster* genomes [27]–[29]. Using recently developed techniques such as ChIP-Seq [30] and RNA-Seq [31], a large amount of data, including the genomic binding data for more than 20 TFs, expression profiles of all protein-coding genes and miRNAs across the developmental time course, as well as refined annotation of 3′UTRs and their regulatory elements in *C. elegans* [32] have been generated. Apart from *C. elegans* and *D. melanogaster*, similar genome-wide datasets in other higher eukaryotes such as human and mouse are emerging. Together with existing information such as protein-protein interactions and miRNAs target prediction in all these organisms, there is an unprecedented opportunity to examine various levels of eukaryotic regulation. Toward this goal, the proper integration of various datasets plays an essential role.

In this study, we propose an integrated network framework for analyzing multi-level regulation in higher eukaryotes, namely the integrated regulatory network. To our knowledge, this is the first attempt to construct system-wide network using experimentally identified TF target genes and miRNAs. More specifically, we identified the target genes and target miRNAs for a set of TFs based on the ChIP-Seq binding profiles. The interactions were then integrated with predicted targets of miRNAs, which are based on annotated 3′UTRs (the 3′UTRome) and conservation information. Making use of the system-wide RNA-Seq profiles, we classified the transcription factors into positive and negative regulators and thus assigned a sign for each regulatory interaction. Protein-protein interactions and a novel intra-regulation between miRNAs using the embedding of miRNAs in their host genes were further incorporated. Leveraging the rich data generated by the modENCODE project, we use *C. elegans* as a primary model to illustrate our formalism and further confirmed our results in human and mouse. As more and more genome-wide ChIP-Seq and RNA-Seq data are generated via the modENCODE and ENCODE project [33] in the near future, the methods of data integration proposed in this work have various potential applications.

## Results

### A general network framework of data integration

**Figure 1:**
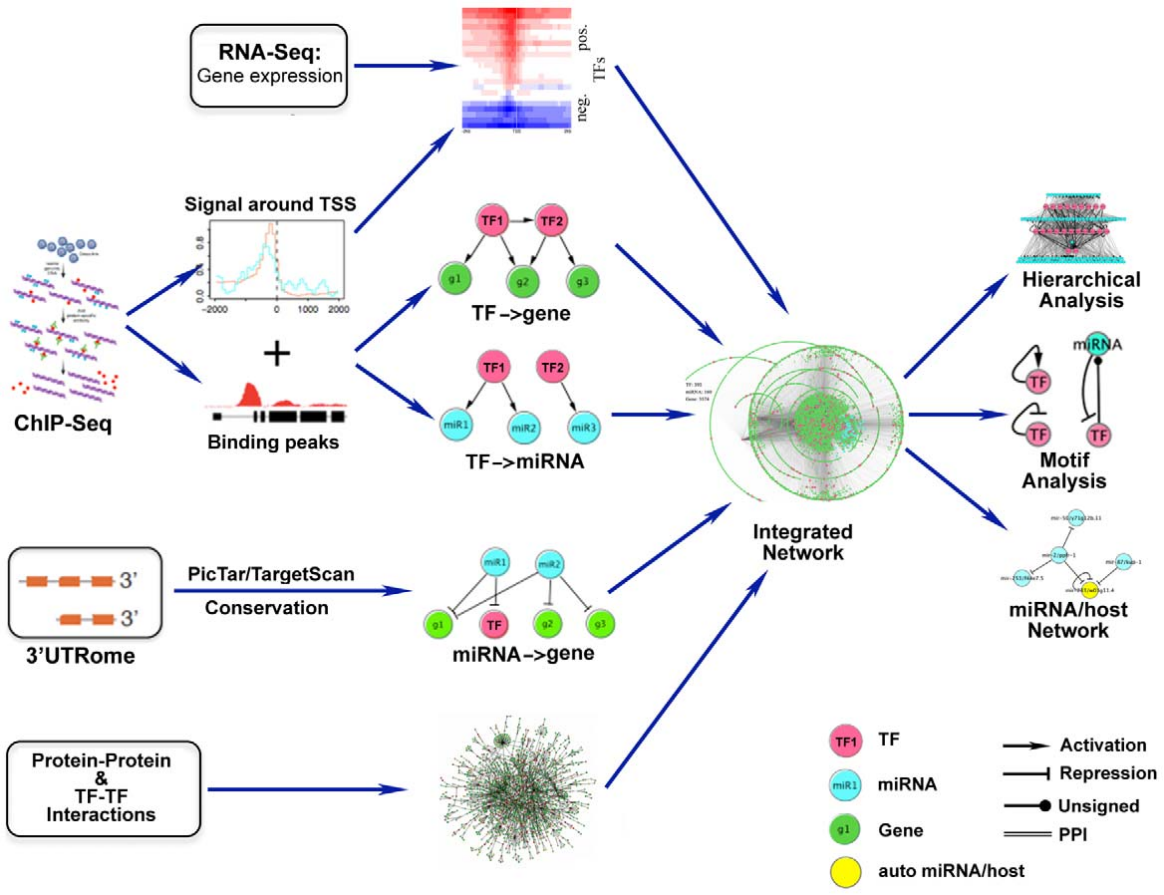
Schematic diagram of the construction and analysis of the integrative regulatory network. ChIP-seq data were used to determine target genes and miRNAs of transcription factors. miRNA target genes were predicted using PicTar or TargetScan algorithms together with conservation information. The three types of regulations form the basic network. The sign of each regulatory interaction was determined based on the correlation between TF binding and gene expression, Extra edges of protein-protein or TF-TF combinatorial interactions were incorporated. We studied the topological structure of the integrated network, including hierarchical organization and motif enrichment.

At the heart of our study is the construction of an integrated regulatory network. The integrated network consists of three major network components: TF-Gene regulatory network, TF-miRNA regulatory network and miRNA-Gene regulatory network. The TF-Gene and TF-miRNA interactions are extracted from ChIP-Seq binding profiles. Predicted targets of miRNAs are identified by the PicTar and TargetScan algorithm [9] using the 3′UTRome, and the predictions are further refined by conservation information. With the basic network in hand, we color the edges in terms of their signs of regulation via expression data, and incorporate extra edges by protein-protein interactions (see Figure 1 for a summary and the Materials and Methods for details).

#### TF-Gene and TF-miRNA regulatory networks

In *C. elegans*, the modENCODE consortium has carried out ChIP-seq experiments for 22 TFs under one or more developmental stages from Early Embryo (EE), Late Embryo (LE), Larva 1 (L1), Larva 2 (L2), Larva 3 (L3), Larva 4 (L4) to Young Adult (YA). Making use of these system-wide binding profiles and the latest annotation, we explored the distribution of TF binding signals around the transcription start sites (TSS) of *C. elegans* genes and found that binding sites of all TFs are enriched close to the TSS (Figure S1). Essentially, a gene is identified as the target of a TF if at least one binding peak of the TF falls within the TSS proximal region (from 1 kb upstream to 500 bp downstream) of the gene. Previous studies have shown that miRNA expression is regulated in a similar manner as protein-coding genes [26], [34], [35]. For example, Martinez et al. have shown that the vast majority of miRNA promoters drive expression with similar activities to that of protein-coding gene promoters. It has also been demonstrated that DNA fragments upstream of the pre-miRNAs are sufficient to initiate their transcription [36]–[39]. Though the TSS of the majority of *C. elegans* miRNAs has not been determined, the starting positions of their corresponding pre-miRNAs are available from the miRBase database [40]. Like protein-coding genes, we observed enriched TF binding signals around these pre-miRNA start positions (Figure S1). We therefore identified the target miRNAs of the 22 TFs in the same way as for protein-coding genes. A miRNA is regarded as the target of a TF if at least one binding peak of the TF falls within 1 kb upstream and 500 bp downstream of the pre-miRNA. Apart from *C. elegans*, we applied the same scheme to identify TF-Gene and TF-miRNA interactions for human and mouse based on ChIP-Seq data for 13 TFs in human K562 cell line, and 12 TFs in mouse embryonic stem cells [41] (see Materials and Methods).

#### miRNA-Gene regulatory network

Predicted targets of miRNAs are identified by PicTar or TargetScan algorithm. In *C. elegans*, we further refined the predictions by taking into account the conservation of miRNA seed sites in three (*C. elegans*, *C. briggsae*, *C. remanei*) or five (*C. brenneri*, *C. japonica* additionally) species. A more detailed description can be found in [42]. Briefly, we identified a total of 20,427 predicted conserved target sites within 4,866 3′UTRs for 2,244 genes. This set of predicted miRNA-mRNA binding sites constitutes a framework for potential interactions underlying miRNA post-transcriptional regulatory networks in *C. elegans*. In human and mouse, miRNA targets are downloaded from [43], [44] and predicted by TargetScan algorithm, which also take into account the conservation of miRNA binding site across multiple mammalian species.

### Basic topology of the *C. elegans* integrated regulatory network

**Figure 2:**
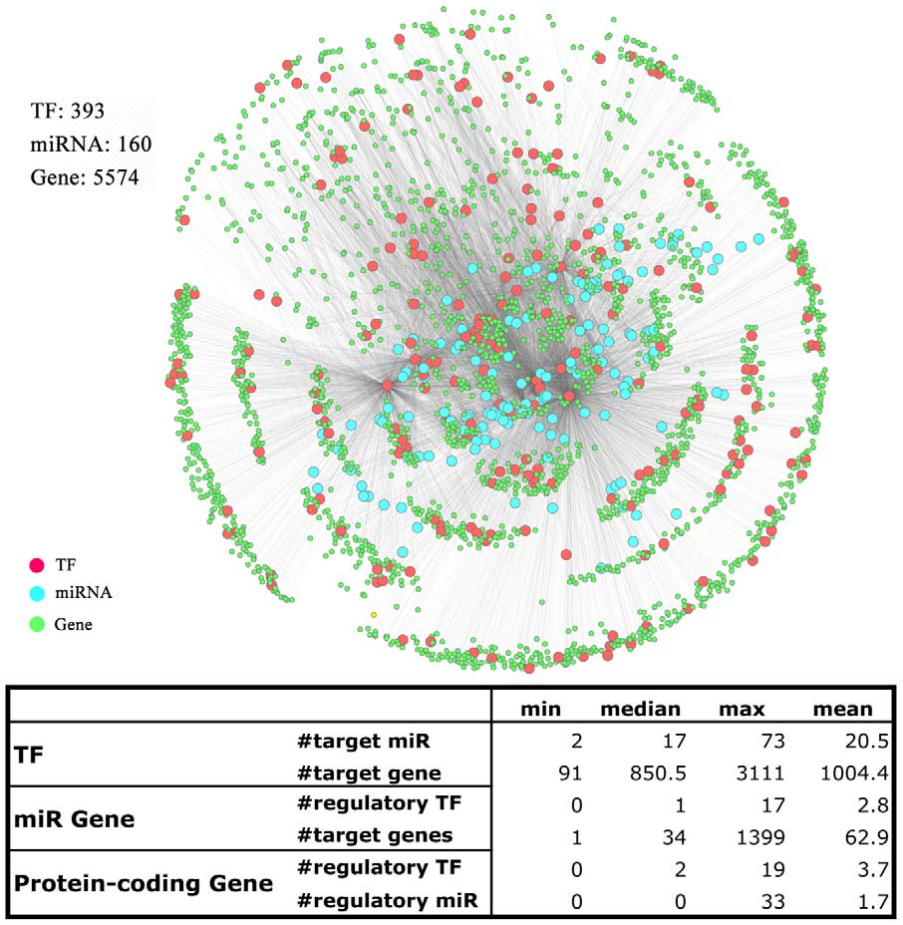
Topology of the integrated regulatory network in *C. elegans*. The network contains 393 TFs (red circles), 160 miRNAs (cyan circles) and 5574 non-TF protein-coding genes (green circles). For 22 of these TFs, we determined the target genes and miRNAs. Topological features of the three node types were shown in the lower table.

The basic integrated regulatory network of *C. elegans* consists of three types of nodes: 393 TFs (among which 22 have target protein-coding genes and target miRNAs available), 5,574 non-TF protein-coding genes and 160 miRNAs. There are 22,096 TF-gene (including TF-TF) interactions, 452 TF-miRNA interactions, and 10,069 miRNA-gene interactions (Figure 2). The number of targets varies dramatically among the 22 TFs, e.g. the number of miRNA targets range from 2 to 73 with a median of 17. Although the difference in target numbers may arise due to experimental parameters such as the sequencing depth and the data quality, it also reflects the biological functions of transcription factors. For the 22 TFs, the number of target protein coding genes and the number of target miRNAs are positively correlated (r = 0.9, P<10^−8^). We compared the number of regulatory miRNAs for TFs with that of non-TFs and found that non-TF mRNAs were on average regulated by 4.6 miRNAs, whereas TF mRNAs were regulated by 6.3 miRNAs. This suggests that miRNAs are more likely to regulate TFs than non-TFs (P = 1.2E-6, Wilcoxon Rank Sum test), which is consistent with previous reports [25].

**Figure 3:**
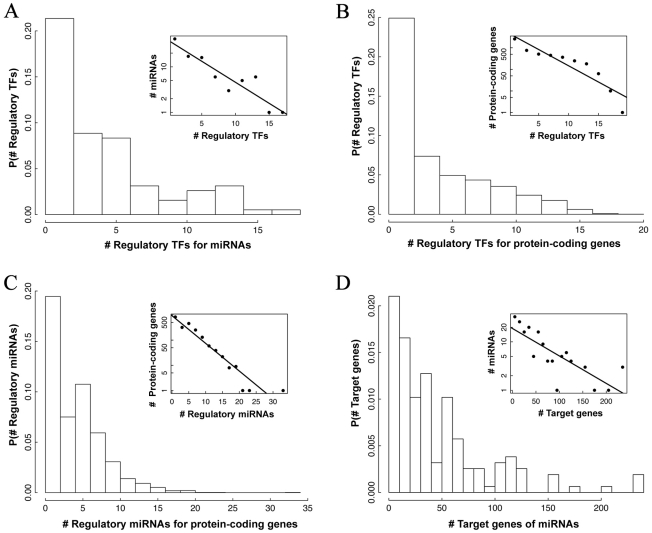
Distributions of the topological features of each node type in *C. elegans* integrated regulatory network. (A) The number of regulatory TFs for miRNAs; (B) the number of regulatory TFs for protein-coding genes; (C) the number of regulatory miRNAs for protein-coding genes; (D) the number of target genes of miRNAs. Each is best fitted to an exponential distribution as shown by the corresponding inset.

To have a systematic overview of the integrated network, we examine the degree distribution of the network. As a result of different types of nodes and edges, there are several kinds of degree distributions (Figure 3). We examined the number of regulatory TFs for miRNAs as well as for protein-coding genes, and found that both are best fitted by an exponential distribution (R^2^ = 0.86, 0.84), implying that a single target gene or miRNA is less likely to be regulated by many TFs simultaneously (Figure 3, top left and right). The number of target genes, and target miRNAs for the 22 TFs, on the other hand, are shown in Table S4. While it is hard to infer the underlying distribution, the number of target genes varies quite a lot. Of particular interest in the integrated network are the miRNA nodes, as they possess both in-degree (the number of regulatory TFs) and out-degree (the number of their target genes). Our analysis indicates that both in-degrees and out-degrees of miRNAs are best fitted by an exponential distribution (R^2^ = 0.95, 0.81) (Figure 3, bottom left and right), which is distinct from the power law distribution exhibited by many other biological networks. However, the maximum in- and out-degrees of miRNAs are 20 and 200 respectively, and are still much larger than expected by chance [25]. We calculated the correlation between in- and out-degrees for miRNAs and found a weak positive correlation (r = 0.2, P<0.01). This is a mathematical indication of loopy structures in the network.

### Combinatorial regulation in the *C. elegans* integrated regulatory network

It has been suggested that combinatorial regulation, the tendency of two or more regulators controlling the same target, plays an important role in transcriptional regulation [45]–[49]. Apart from the case of two TFs, the combinatorial effects of a TF-miRNA pair have recently been addressed [24], [50], [51]. To explore this combinatorial regulation via the integrated network in *C. elegans*, we examined the tendency of sharing common protein-coding targets between 22 TFs and 160 miRNAs. Many TF-miRNA pairs show significant target overlap in a hypergeometric test, which are presumably responsible for the same function (Figure S2). Similarly, we quantified for each possible pair of TFs, the tendency of sharing common protein-coding targets (Figure S3) and common miRNAs (Figure S4), and found many significant pairs.

### Hierarchical analysis of the *C. elegans* integrated regulatory network

**Figure 4:**
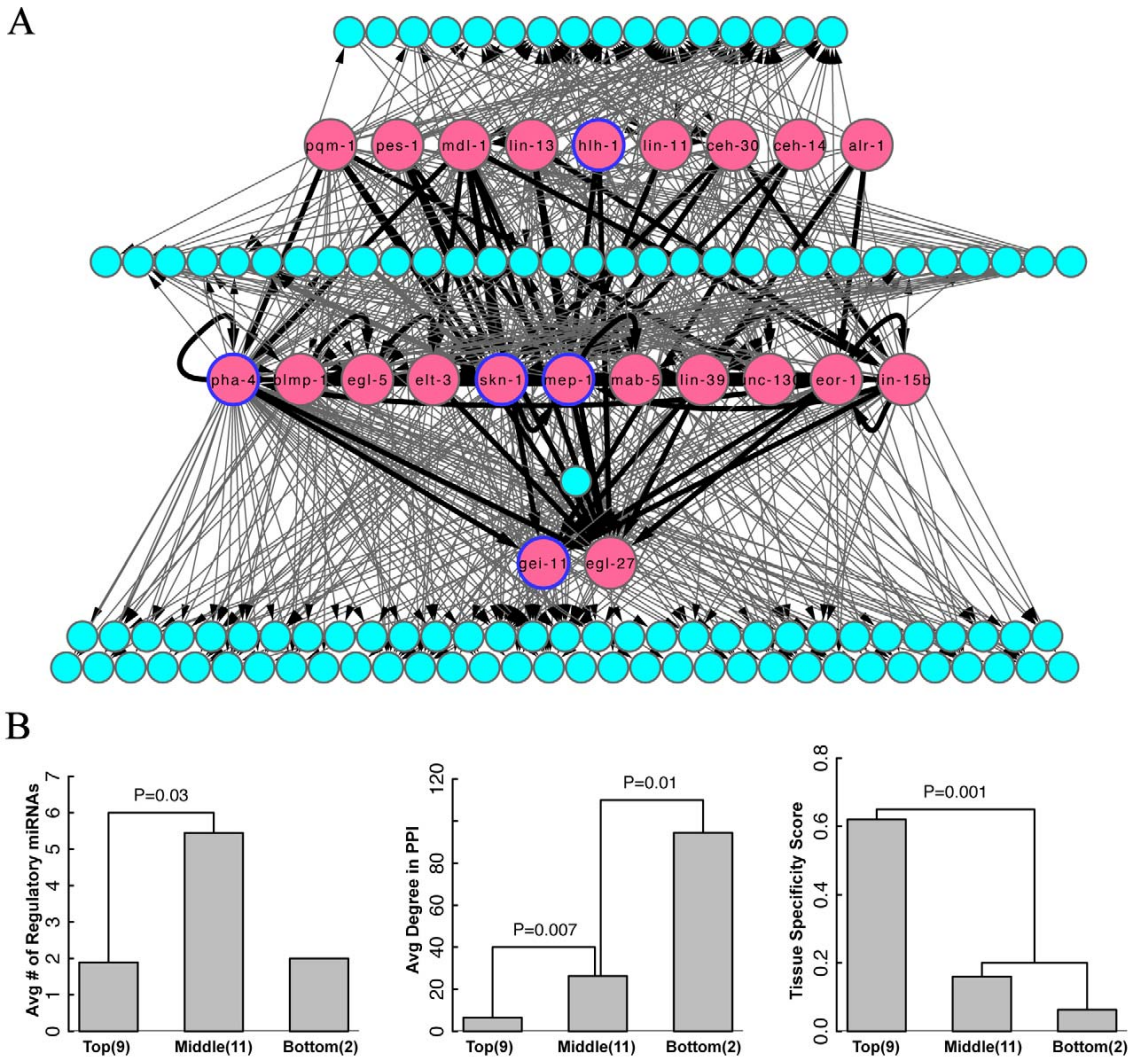
Hierarchical illustration of the integrated regulatory network. (A) The *C. elegans* integrated gene regulatory network exhibits a 7-layer structure with 3 layers of TFs (red circles) and 4 layers of miRNAs (cyan circles). TF-TF and TF-miRNA regulatory interactions were shown as dark and light arrows respectively. Essential transcription factors are labeled by a blue circle. (B) TFs in the three layers show significant difference in their average number of regulatory miRNAs (left), average degree in protein-protein interaction network (middle) and tissue specificity (right).

To better visualize the regulatory interactions in an integrated regulatory network, we built an intuitive hierarchy comprising of TFs and miRNA that would allow a clear mining of underlying regulatory association between various regulators. A conventional hierarchy requires all regulatory interactions to point down in the hierarchical structure; no regulators regulate those above them. This requirement might pose problems in the presence of cycles in the network, which is the case when miRNA are included in the integrated network. To overcome this problem, we used only the transcriptional regulatory interactions to first build a core hierarchy strictly following “chain of command” pointing down as used in previous studies (see Materials and Methods) [17]. In *C. elegans*, this approach results in 3 layers of TFs with 9 at the top, 11 in the middle and 2 TFs in the bottom layer, respectively (Figure 4A). The interactions involving the miRNAs were then added to this core hierarchy to build the integrated hierarchy.

The importance of hierarchical analysis is signified by the fact that TFs at different levels are found to have different characteristics. We correlated the hierarchical levels of the 22 TFs with various functional genomics data (see Table S1), and observed several features that are significantly different between TFs from different levels. First of all, we found that TFs downstream of the hierarchy are more likely to be essential, whereas those at the top are likely to be non-essential (statistically, this result is not significant due to small sample size). More specifically, while 5 out of the 22 TFs are experimentally verified to be essential for the survival of the *C. elegans* according to RNAi screening [52], four of them are in the middle or the bottom layers, and only one is in the top layer. Secondly, we found that the TFs in different layers possess different topological properties in the *C.elegans* protein-protein interaction network. In particular, the average numbers of interaction partners for TFs in the top, middle and bottom layers are 6, 26 and 95 respectively (Figure 4B). Thirdly, we calculated and compared the tissue specificity of TF at the three layers in 8 different tissues (see Materials and Methods) and found that those lower layer TFs are more uniformly expressed in these tissues (Figure 4C). Finally, of particular interest is the number of miRNA regulations targeting the three layers. We found that of the three layers, TFs in the middle layer are more likely to be regulated by miRNAs (Figure 4D). The hierarchical network is constructed to make TFs at higher layers regulating those at lower layers, thus higher layer TFs might also have more target genes and miRNAs.

We examined the correlation between other properties of TFs and their corresponding levels, including their expression, conservation information, stage specificities (see Materials and Methods) and their target miRNAs across the worm developmental time course. In our analysis, these properties did not show significant differences between the three layers. However, some of them were reported to be significant in the hierarchical network in yeast [53].

### Positive and negative regulators in the *C. elegans* integrated regulatory network

**Figure 5:**
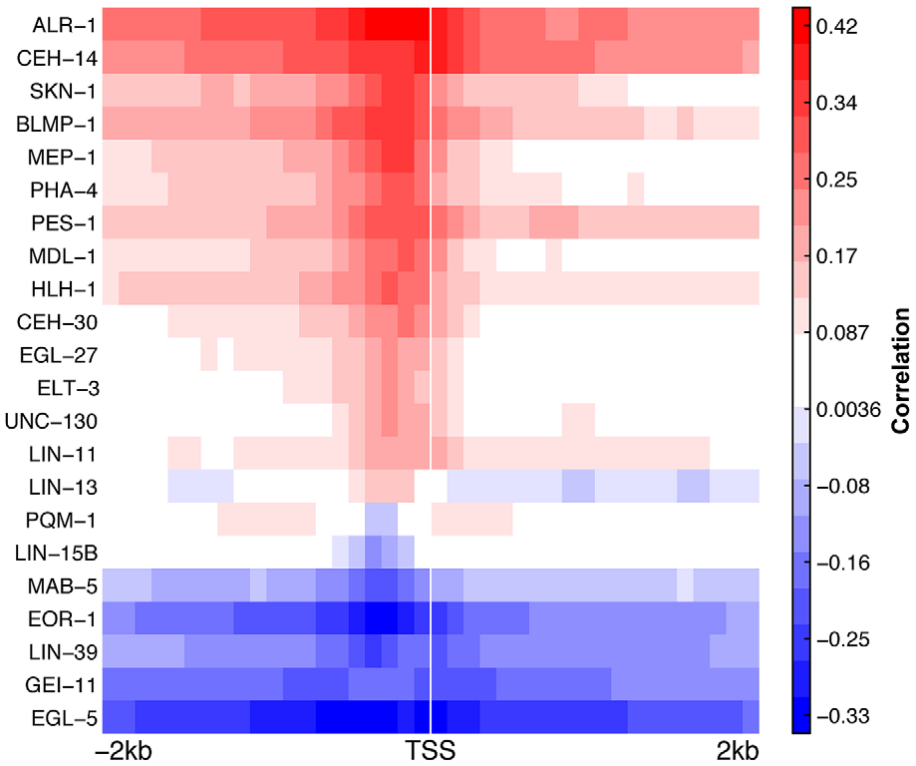
Correlation of gene expression with TF binding signals in DNA regions around transcription start site (−2 kb∼2 kb). Based on their correlation patterns, TFs were divided into positive (red) and negative (blue) regulators.

While the integrated network we constructed describes the target genes and miRNAs of TFs, the kind of the regulatory interactions are not known. To provide further insights, we examined for each TF, the correlation between the binding signals around the TSS and the corresponding target gene expression (see Materials and Methods for details). As shown in Figure 5, in *C. elegans,* many TFs show either a consistent positive or negative correlation from −2 kb to +2 kb of the TSS. We therefore classified the 22 TFs into two classes: positive regulators (e.g. ALR-1, CEH-14) and negative regulators (e.g. EGL-5 and EOR-1). With the assignment of positive and negative regulators, an edge in the network pointing from one of the 22 TFs is regarded either as a positive edge or a negative edge, depending on the class of the TF. In addition, we regarded regulatory interactions by miRNAs as negative, due to the very nature of miRNAs [54]. As a result, all of the 32,617 regulatory interactions in the integrated regulatory network of *C. elegans* were assigned with signs.

### Network motifs in the *C. elegans* integrated regulatory network

**Figure 6:**
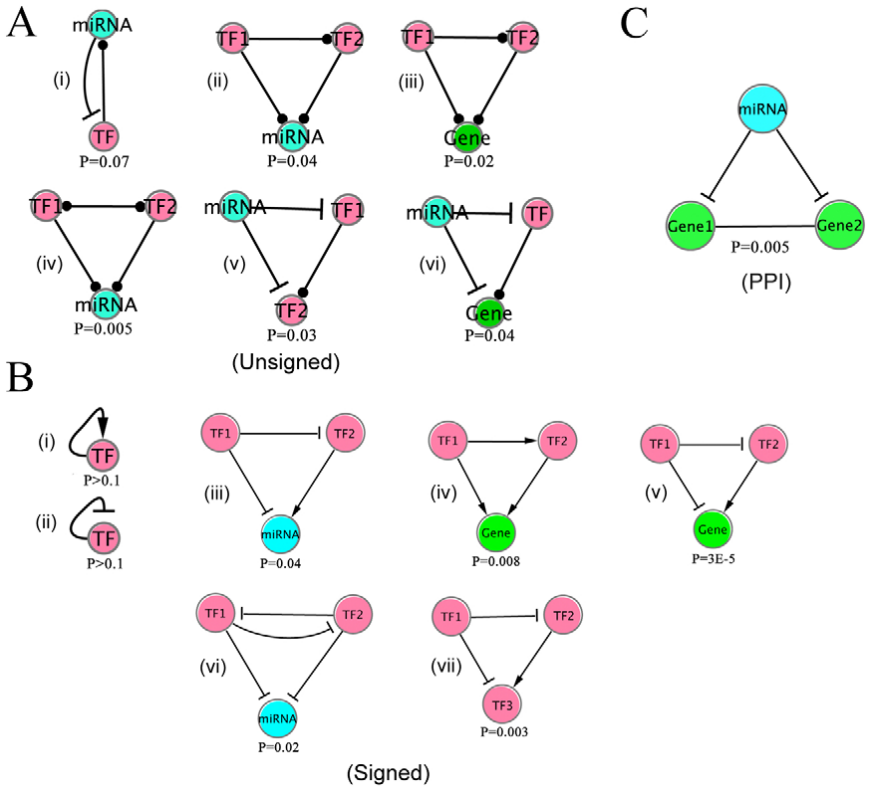
Representative network motifs in integrated gene regulatory network for *C. elegans*. (A) motifs in the unsigned network. (B) motifs in the signed network. (C) a composite motif in which a miRNA represses two physically interacting genes. P-values are calculated by comparing the number of occurrences of each motif in the real network with those in random networks.

Previous studies suggest that network motifs, a set of recurring patterns originally defined in transcription regulatory networks, are responsible for carrying out specific information-processing functions. Moreover, studies on network motifs have found that motifs with the same geometrical structure but different signs of regulation could have profound differences in terms of functions [23]. Here we categorized several motifs in the *C. elegans* integrated regulatory network (Figure 6).

A transcriptional auto-regulatory feedback loop is the simplest network motif built out of a transcription factor regulating its own transcription (Figure 6B, (i) and (ii)). Among the 22 TFs, we identified 6 auto-regulated factors: ELT-3, PHA-4, UNC-130, EGL-5, LIN-15B and MAB-5. However, there is no evidence to show that auto-regulation is over-represented in our data set (P>0.1, permutation test), probably due to the small number of TFs. We further divide the auto-regulators into negative auto-regulation (EGL-5, LIN-15B and MAB-5) if the TF is a repressor and positive auto-regulation (ELT-3, PHA-4 and UNC-130) if it is an activator [23]. In general, positive regulators (PAR) reinforce a signal while negative auto-regulators (NAR) stabilize a system. Both of the NAR and PAR have been frequently reported in previous studies [55]–[58]. Particularly, the NAR motif occurs in about half of the repressors in *E. coli* [59], and in many eukaryotic repressors [11].

In the integrated regulatory network, there are 452 TF→miRNA regulatory relationships and 81 miRNA→TF regultory relationships. It has been shown that the TF⇔miRNA composite feedback loops (a TF that regulates a miRNA is itself regulated by that same miRNA) occur more frequently than expected by chance in *C. elegans* (Figure 6A, (i)). Without taking signs into account, we identified 15 TF⇔miRNA miRNA composite feedback loops (see Table S2) from our integrated network, which is moderately over-represented (P = 0.07, permutation test).

We extensively constructed all 3-node sub-graphs (see Figure S5 and S6) in the integrated regulatory network, and compared their occurrence with what would be expected in an ensemble of random integrated networks. The counting of different sub-graphs and network randomization were performed by a sampling tool called FANMOD [60], [61] (see Materials and Methods for details). Without considering the signs of interactions, we found a set of 5 over-represented 3-node motifs in the integrated network (Figure 6A). Motif A (iii) is the traditional transcription factors mediated feed-forward loop (FFL), which is known to be enriched in the transcriptional regulatory networks of organisms like yeast and *E. coli* [62]–[64]. Motif A (ii) is similar to motif A (iii) except the target gene is replaced by a miRNA. Motifs A (v) and A (vi) are novel, and they share a common construction feature in which a miRNA regulates a TF as well as its downstream target. We then repeated the procedures with signs taken into account. Figure 6B demonstrates a list of enriched motifs in the integrated network with the signs taken into consideration. Motif B (iv) is the well known coherent type 1 FFL [20]. B (iii), B (v) and B (vii) share a common design structure: a TF as well as its downstream target (gene, TF or miRNA) are simultaneously repressed by a common TF. Interestingly, these motifs are all coherent in the sense the indirect path has the same sign as the direct path. B (vi) is a composite motif that consists of a toggle switch formed by a pair of mutually repressing TFs, and both TFs repress a common miRNA. In principle, both enriched and depleted motifs are worth studying, however, no significantly depleted motif was found in our network.

### Other levels of microRNA-coordinated regulation

The integrated regulatory network we constructed has demonstrated how miRNAs coordinate the transcriptional activities. To systematically explore the coordination of cellular activities by miRNAs, we extended our study to two other levels of miRNA-mediated regulations.

First, miRNAs regulate protein complexes by regulating their individual components. Systematically, these could be examined using various genome-wide protein-protein interaction (PPI) networks. We studied the regulation in *C.elegans* using a PPI network downloaded from Worm Interactome Database [65] (see Materials and Methods for details). The network contains 6,125 nodes and 177,267 edges. From the level of individual proteins, we correlated the degree in the PPI network with the number of regulatory miRNAs. The results indicate that miRNAs tend to regulate hub genes in the PPI network, agreeing with previous observation by Liang et al [66]. In addition, the same pattern is observed in the transcriptional regulation of hub genes. For instance, the genes with degree >20 are on average regulated by 1.32 miRNAs, significantly greater than genes with degree ≤20, which on average have 0.95 regulatory miRNAs (P = 0.004, Wilcoxon Rank Sum test). On the other hand, the same set of PPI hubs are regulated by 3.40 TFs, significantly higher than the rest, which are regulated by 2.03 TFs (P = 2E-6, Wilcoxon Rank Sum test). Apart from the level of individual proteins, we studied how interacting proteins are collectively regulated by a miRNA by introducing an additional type of edge (protein-protein interaction) to the integrated gene regulatory network. We found that, compared to a randomized network with the same degree distribution, interacting proteins in the PPI network are more likely to be regulated by the same miRNAs (P = 10^−7^). In other words, we observed another interesting motif with a pair of interacting proteins being regulated by a common miRNA (Figure 6C) [67].

**Figure 7:**
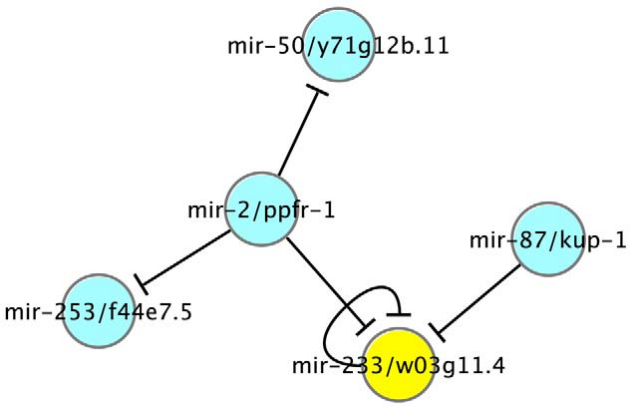
Intra-regulation among miRNA/host-gene pairs in *C. elegans*. The regulatory relationships among the 39 miRNA/host-gene pairs (the miRNAs are embedded within the intron of the host in the same sense orientation) form a small miRNA-host network consisting of 5 interactions. The auto-regulated mir-233/w03g11.4 was highlighted in yellow color, for which mir-233 is predicted to repress the expression of its host-gene, w03g11.4.

Secondly, the embedment of miRNAs in their host genes hinges at a novel intra-regulation between miRNAs. In *C. elegans*, 60 miRNAs are embedded within the intron of a protein-coding gene (see Table S3), of which 39 are in the sense orientation (P = 0.007). These miRNAs are likely to be co-transcribed with their host gene [6], [68]. We examined the regulatory relationship between the miRNAs and their host gene. The regulatory relationships among the 39 miRNA/host-gene pairs form a small miRNA-host network consisting of 5 interactions (Figure 7). In the network, a directional edge indicates a regulatory relationship from a miRNA to the host gene of another miRNA (possibly itself). As shown in Figure 7, mir-2 represses the host genes of three other miRNA including mir-233; and the host gene of mir-233, W03G11.4, is subject to repression by mir-233 itself, mir-2 and mir-87.

### Integrated regulatory network in human and mouse

**Figure 8:**
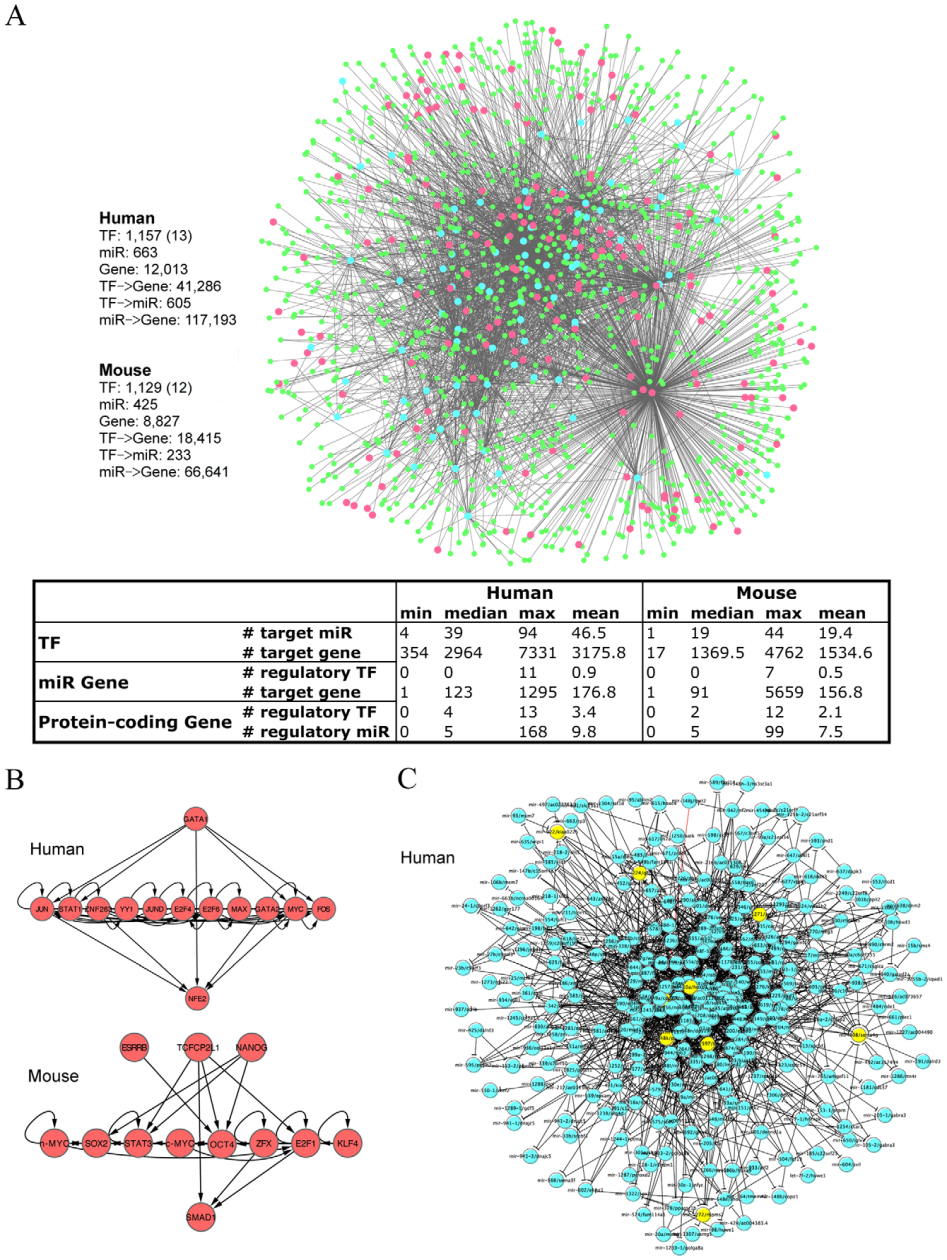
Integrated regulatory networks in human and mouse. (A) Basic statistics. (B) Hierarchical organization of TFs in human and mouse. (C) the miRNA-host network in human. There are 1,426 interactions with 8 auto-regulated miRNA/host-gene pairs (yellow).

So far we have focused on *C.elegans* using the data from the modENCODE project. As similar data of other species is accumulating, it is worthwhile to apply our data integration approach to various systems like human and mouse. Toward this end, we have gathered system-wide ChIP-Seq profiles of 12 mouse TFs and 13 human TFs, and compiled the integrated regulatory networks for both mouse and human (see Materials and Methods for details). Figure 8A shows the details of these networks. Similar to *C. elegans*, the transcription factors in human and mouse can be arranged in a hierarchical fashion (Figure 8B). As the number of TFs sampled is too small, it is however not practical to perform correlation analysis similar to ones in *C. elegans*.

To explore the novel intra-regulation between miRNAs, we constructed a miRNA-host network for human miRNAs. Out of the 939 human miRNAs, 588 overlap with a protein-coding gene. Among them, the majority (482, P = 2×10^−58^) is located in the sense strand of the host gene, resulting in 482 miRNA/host-gene pairs. As we did in *C. elegans*, we identified 1,426 regulatory relationships among these miRNA/host-gene pairs, including 8 auto-regulated pairs (Figure 8C).

**Figure 9:**
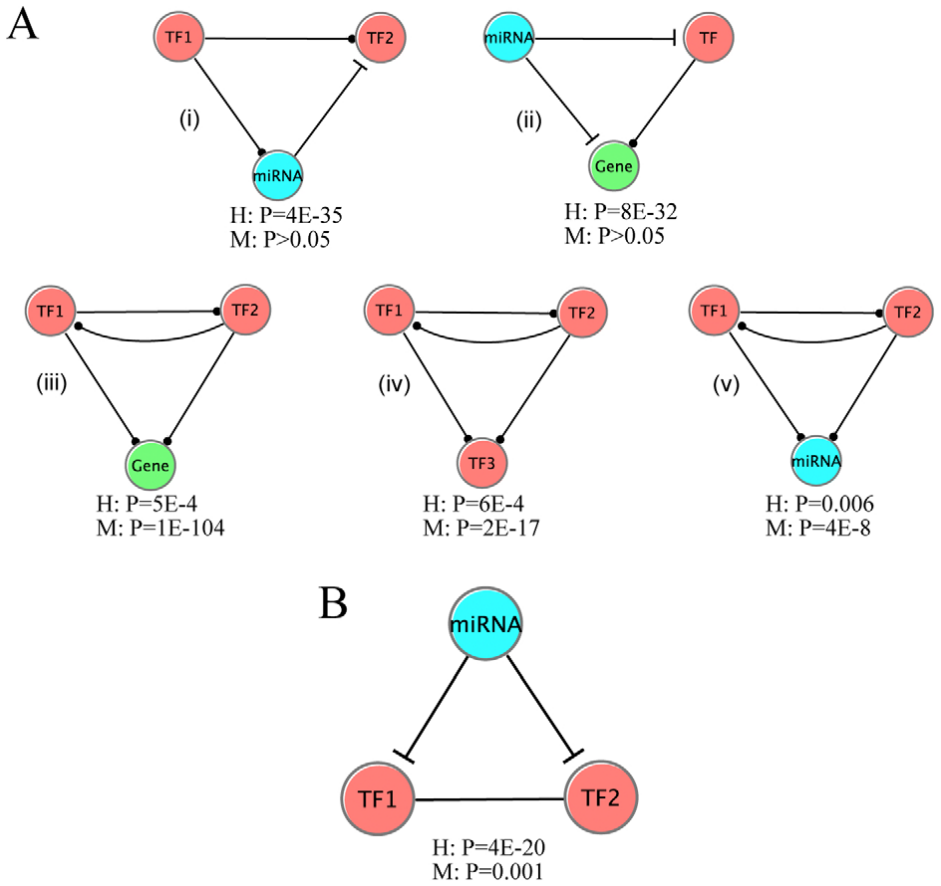
Representative network motifs in the integrated regulatory network for human and mouse. (A) Significant motifs in the regulatory networks. (B) A significant motif enriched in the networks with further incorporation of TF-TF physical interactions. The significances of each motif in human and mouse were shown.

We performed the same motif analysis on the human and mouse integrated regulatory networks (Figure 9). In fact, the integrated regulatory networks of human and mouse share common motifs with *C. elegans*. For instance, Motifs 9A (ii) and (v) are equivalent to Motifs 6A (vi) and (iv) in *C. elegans*. In addition, we found another interesting miRNA mediated feed-forward loop in the human integrated regulatory network (Figure 9A(i)), which has already been reported in literature [69]. As the number of TFs sampled in these systems is far from complete, one should not expect that the results are entirely representative.

Using the recently published human transcription factor physical interaction network and the mouse transcription factor physical interaction network [48], we found that a single miRNA tends to co-regulate a pair of interacting TFs more frequently than by random (P = 4×10^−20^ for human and P = 10^−3^ for mouse). This motif (Figure 9B) is shared in *C. elegans* (Figure 6C). This indicates that miRNAs prefer to coordinately repress physically interacted transcription factors, which might be involved in combinatorial regulation of gene transcription.

### Sensitivity to selection of various parameters

At the heart of our study is the determination of TF-gene and TF-miRNA interactions from ChIP-Seq profiles. The number of interactions obviously depends on the choice of promoter regions, and the inclusion/exclusion of the so called HOT regions [42] (see Materials and Methods for details). While the results presented are based on the exclusion of HOT regions, and a choice of promoter region defined as 1 kb upstream to 500 bp downstream of the TSS for protein-coding genes or of the start position for the pre-miRNAs, one could include the HOT regions to increase statistical power or shorten the definition of promoter region (500 bp upstream to 300 bp downstream) for higher specificity. Moreover, the number of false positives in the miRNA target prediction can be reduced by increasing the conservation of miRNA binding sites from 3 species (*C. elegans*, *C. briggsae*, and *C. remanei*) to 5 species (including also *C. brenneri*, *C. japonica*). To test the robustness of our network motif analysis, we explored the influence of these choices and their combinations. We tested all the possibilities, resulting in a total of 8 integrated networks. Our analysis indicates that these integrated networks are similar in their topology and in presence of over-represented network motifs in spite of the difference in the number of interactions (Table S4).

The fact that the number of regulatory interactions depends on the choice of parameters might lead to a possible drawback, namely the assignment change of hierarchical levels in our hierarchy analysis. Even though the precise assignment of layers indeed changes, the signifying characteristics of different layers remain robust. For example, based on 18 larva TFs (with other 4 embryonic TFs excluded), we have constructed another hierarchy using more stringent parameters: promoter regions defined from 500 bp upstream to 300 bp downstream, excluding TF binding peaks overlapped with HOT regions, and using 5-way conservation for miRNA target prediction [42]. In addition, we further filtered the regulatory interactions whose correlation between the TF and the target gene are weak across different developmental stages. The resultant hierarchy consisted of 9 TFs in the top layer, 4 TFs in the bottom layer and 5 TFs in the bottom layer. Although these numbers differed from those in Figure 4, the overall statistical properties of the two hierarchies are highly consistent. For instance, in the network three essential TFs are in the bottom layer, one in the middle layer, and none in the top layer. Moreover, the TFs in the middle and bottom layers have significantly more physical interactions than those in the top layer (P = 0.002).

As done in other studies based on miRNA target prediction, one should take into account the effects of the choice of different prediction methods. While the target genes of miRNAs shown were mainly identified by using the PicTar algorithm [9], we have also employed the TargetScan algorithm [70]. A comparison of the results between the two algorithms revealed that PicTar identified 99% of seed sites predicted by TargetScan, and conversely, TargetScan identified 89% of seed sites predicted by PicTar, when only the conserved seed sites were considered. It has been demonstrated previously that TargetScan and PicTar are most popular performers for target prediction of miRNAs and generally produce the highest overlap with experimentally determined sites [71], [72]. Thus, the results based on PicTar algorithm were finally used for determining miRNA target genes.

We next examined the sensitivity of the network motif analysis to removal of regulatory interactions. Specifically, we randomly removed 1, 5, 10, 20, 30% of edges (TF→gene, TF→miRNA or miRNA→gene interactions) from the integrated network, and re-performed the network motif analysis. We obtained similar results with the original integrated network. We also examined the effect of excluding one or more of the 22 TFs, namely, removing all the genes and miRNAs targeted by a selected TF. In this case, some of the motifs were not significant, particularly when a TF with large number of target genes/miRNAs was excluded. For these network motifs, a regulatory network containing target information for more TFs would be helpful to gain further confidence on their significance in transcriptional regulation.

## Discussion

In this study, we have presented an integrated network framework for analyzing multi-level regulation in higher eukaryotes, and applied the methods using high-throughput data from *C. elegans*, human and mouse. Our framework makes use of the ChIP-Seq binding profiles of TFs, RNA-Seq expression profiles, 3′UTRome and protein-protein interactions. Several recent genome-scale studies that have attempted to integrate regulation by TF and miRNAs are limited in terms of their datasets. For instance, the work by Yu et al. [24] on human were mostly based on computational predictions, and while Martinez et al. provided experimental TF→miRNA interactions in *C.elegans* based on Y1H assays [26], the regulatory interactions between TF and genes were not incorporated. Neither of these studies considered the signs of the regulatory interactions. Though the number of TFs we used is still relatively small, we believe that our study serves as a first attempt for a comprehensive analysis of multi-levels gene regulation. As more and more data of these types emerge, the methods of integration will play an essential role to decipher the complexity of regulatory network. Our framework can potentially be improved by including reported regulatory interactions from database and literature, by filtering out low confidence interactions, and by including computationally identified regulatory interactions. For example, the existence of TF binding motifs in ChIP-Seq peaks has been examined for improving TF target identification [73]. With the accumulation of the relevant information in *C. elegans*, we would expect a more comprehensive integrated regulatory network in the future.

An interesting observation from our hierarchical analysis in *C. elegans* network is the fact that TFs at lower levels are more likely to be essential and have more interaction partners in the protein-protein interaction network. This observation is consistent with the work by Yu et al. in yeast [53]. Yu et al. suggested that the middle or bottom layer TFs play the role of “mediators” or “effectors”, and thus require more intensive information exchange with other proteins. These TFs are more likely to be in charge of the fundamental cellular processes, and therefore certain pathways will cease operating upon their deletion, causing a lethal effect. The top layer TFs, on the other hand, act more like “modulators” which coordinating gene expression across different pathways. Even though the inhibition of these TFs affects the precise expression among pathways, most of the pathways remain functional and therefore the organism can survive. Of particular significance is the degree of validity of the design principle in yeast for multi-cellular organisms such as *C. elegans*. Interestingly enough, TFs at the bottom have lower tissue specificity, i.e. they are expressed in many tissues. This observation is consistent with the fact that the bottom TFs are in charge of the fundamental cellular processes. Our analysis hinges at a close similarity in the hierarchical organization of transcriptional regulatory network in yeast and higher eukaryotes such as *C. elegans*.

The hierarchical layout as shown in Figure 4 suggests another design principle in multi-level genetic regulation, namely miRNAs tend to regulate TFs in the middle of the hierarchy. As observed separately based on transcriptional regulatory networks, protein modification networks and phosphorylation network in Ref. [17], regulators at the middle level are responsible for the proper organizational effectiveness, and thus they have the highest collaborative propensity and co-regulator partnerships. Our result suggests that, the same principle is also true for different types of regulations in an integrative picture.

We have identified several over-represented network motifs in the integrated regulatory network, including the well known transcription factors mediated feed-forward loops. The coherent FFLs share a common design structure, suggesting that both protein-coding genes and miRNAs are regulated by a pair of transcription factors in a similar fashion. Of particular interest are the miRNA-mediated motifs in which miRNA regulatory interactions are employed. For instance, we found 15 composite TF⇔miRNA feedback loops. The same motif was reported to be more frequent than expected by chance in [26]. While feedback loops are rare in pure transcriptional regulatory networks [19], [20], the enrichment of composite feedback loops suggests that feedbacks are more likely to involve multiple levels. It has been discussed in Ref. [26] that, in a composite feedback loop, the sign of the transcriptional regulation determines the function of the loop. A loop with a transcriptional repressor works as a bi-stable switch and a loop with a transcriptional activator can function as a steady state or an oscillatory system. Interestingly, among the 15 composite feedback loops we observed, there are 6 transcription factors involved and all of them are activators. It is therefore more reasonable that the composite feedback loops we observed are responsible for ensuring robustness during development [51] or playing a role in periodic processes such as molting in different larval stages.

Previous studies [25] and ours have reported that miRNAs tend to regulate transcription factors. As shown in our motifs analysis, instead of targeting individual transcription factors, miRNAs tend to regulate transcription factors as well as their downstream targets. By shutting down a gene together with its transcriptional activator, the motifs could be viewed as an effective strategy to shut down the target gene in a longer time period. A similar motif is observed in the protein-protein interaction network, in which a miRNA tends to target a pair of interacting proteins, presumably comprise a molecular machine. From one point of view, the motif is an effective way to shut down a function. The production of certain useless components is considered wasteful, and they would lead to various promiscuous interactions in the cell. On the other hand, the removal of the unwanted components might increase the response time of the cell when the machine is in need. Therefore, the usage of such motif depends on the production rate of the components. For instance, if a component of the machine requires a longer time to produce than the others, the shut down of every component at the same time might not be very effective. Nevertheless, the two miRNA-mediated motifs described are also found in mouse and human.

We have explored a novel mechanism in which miRNAs might regulate one other via their host genes, involving 5 miRNAs and 5 interactions. Though this is a relatively small number compared to the total number of miRNAs in the genome, it is intriguing that the 5 interactions are not separated but connected to form a small network, suggesting that the interactions may have real biological significance. Among the 39 miRNAs that are embedded in the same sense within the intron of a host gene, we found only one case in which the miRNA (mir-233) is being regulated by itself, i.e. mir-233 has a conserved binding site in the 3′UTR of its host gene (W03G11.4). This suggests that the repression of a miRNA on its host is not desirable and thus tends to be eliminated from the genome. A similar but more comprehensive analysis performed using human data points to the same conclusions. It is worthwhile to point out that in this mechanism, the target miRNA might not always be down regulated since miRNAs typically function by degrading the target mRNAs or by inhibiting their translation [6].

## Materials and Methods

### Datasets and gene annotation

The binding sites of ∼30 *C. elegans* TFs were determined using ChIP-Seq experiments. The data sets were examined manually to remove experiments with low read mapping rate, small number of calling peaks, or low reproducibility between replicates. After removing these low quality experiments, we finally obtained the binding data sets for 22 TFs. The binding signals for all TFs were normalized against background signals measured using the corresponding input DNA samples. The binding peaks were identified using the PeakSeq method [74]. More detail information about the ChIP-seq assay and data pre-processing has been previously described in [75]. The list of the 22 TFs and their features can be found in Table S1. Expression levels for all annotated worm transcripts at different developmental stages were quantified using RNA-seq [76]. MicroRNA expression levels at different developmental stages of *C.elegans* were obtained from small RNA-seq measurements performed by Kato et al. [77]. All these data are available from the modENCODE website at http://www.modencode.org.

The *C. elegans* protein-protein interaction data were downloaded from the Worm Interactome Database [65]. The data contain 178,152 interactions that are determined by yeast-two-hybrid experiments, literature curated or by computational analysis. Annotation of worm transcripts was downloaded from WormBase at [78] or from the Ensembl database at http://uswest.ensembl.org/index.html. Annotation of nematode microRNAs was downloaded from the microRNA database miRBASE at http://www.mirbase.org [40]. Assembly version WS180 of *C.elegans* was used for gene and microRNA annotations as well as for data processing.

The TF-TF interaction data set was downloaded from Ravasi et al. [48]. The data set contains 762 and 877 interactions in human and mouse, respectively. Annotation for human and mouse Refseq genes was downloaded from UCSC Genome Browser at http://genome.ucsc.edu/. Annotation for human and mouse miRNAs was based on miRBase [40]. ChIP-Seq experiments for 12 mouse TFs in embryonic stem cells were performed by Chen et al [41]. These TFs are E2F1, ESRRB, KLF4, NANOG, OCT4, STAT3, SMAD1, SOX2, TCFCP2L1, ZFX, c-MYC and n-MYC. ChIP-Seq data for 14 human TFs in K562 cell line, E2F4, E2F6, FOS, GATA1, GATA2, JUN, JUND, MAX, MYC, NFE2, STAT1, YY1 and ZNF263, were generated by the ENCODE project and are available from UCSC Genome Browser.

### Identification of target coding and non-coding genes for TFs

We identified the target protein-coding genes and miRNAs of the 22 TFs based on the ChIP-Seq binding data sets. DNA regions with the binding peaks were potential targets of the transcription factor. We observed that 304 specific DNA regions, about 400 bp in size, were bound by 15 or more factors; we termed these regions the Highly Occupied Target (HOT) regions. We found that the binding motif of each individual TF is not highly enriched in these HOT regions, suggesting that the TFs are not directly associated with DNA via specific binding sites. These HOT regions therefore were not regarded as the targets of transcription factors. To identify the list of targets, we obtained the annotations of 27,242 worm genes from Ensembl database at http://uswest.ensembl.org/index.html. A gene was considered as the target gene of a TF if the center of at least one binding peak of the TF followed into the promoter region (1000 bp upstream and 500 bp downstream of the TSS) of the gene. Similarly, a miRNA was referred as the target of a TF if at least one peak is found around the start position of the corresponding pre-miRNA (1000 bp upstream and 500 bp downstream of the TSS). The 1000 bp upstream to 500 bp downstream criteria were determined according to the binding signal distribution of TFs around the TSS. We found that >80% binding signals were restricted to these 1.5 kb-DNA regions for most TFs. Other criteria can also be used to obtain stricter (500 bp upstream to 300 bp downstream) or relaxed (2000 bp upstream to 500 bp downstream) target gene sets.

### Prediction of miRNA target genes

PicTar algorithm [9] was applied to a well-defined set of 3′UTRs [32] to identify miRNA target sites. To reduce false predictions, we considered only the miRNA target sites that are conserved across three (*C. elegans*, *C. briggsae*, and *C. remanei*) or five (*C. brenneri*, *C. japonica* additionally) species. The binding sites for all of the 174 annotated miRNAs in miRBase [40] were identified. A gene was considered a target of a miRNA if there was at least one conserved binding site in the 3′UTR of at least one transcript of the gene. More details have been described in [42].

### Construction of integrated gene regulatory network

The experimentally identified TF→gene and TF→miRNA interactions were combined with predicted miRNA→gene interactions to form an integrated gene regulatory network. In the network, we only included the genes for which both the TF binding data and miRNA target site prediction were available, namely, the genes used as the input for TF target identification and miRNA target prediction.

### Identification of enriched motifs

Enriched motifs were identified by the software FANMOD [60]. Instead of counting the occurrence of a certain motif, the software estimates the occurrence frequency (N_real_) via sampling, and the number is compared to the frequencies of an ensemble of 1000 random networks. The set of random networks is generated by FANMOD (with default parameters), in which the edges are rewired while keeping its rough topological statistics constant. Specifically, the null models are generated by a Monte Carlo type algorithm which rewires the original network while keeping the same number of coding gene targets and the number of miRNA targets for a TF node, the number of targets for a miRNA node, and the number of regulatory TFs and miRNAs for a gene node [79]. The occurrence frequencies of a motif in the ensemble follow a Gaussian, and the enrichment of the motif is quantified by a 
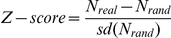
. For the signed integrated regulatory network, the set of random networks is generated by rewiring such that, apart from the number of each type of regulatory interactions in each node is preserved, the number of positive and negative regulations in each node are separately preserved.

### Identification of positive and negative regulators

We therefore divided the DNA region from 2 kb upstream to 2 kb downstream of the TSS of each transcript into 40 small bins, each of 100 bp in size. For each bin, we calculated the average signal of each TF binding profile across all transcripts. Specifically, the number of reads that cover a bin was counted and weighted according to their overlap with the bin. We then calculated for each TF in each bin, the Pearson correlation between the average signal and the expression of the corresponding transcripts. A consistent positive (negative) correlation across the bins means that the TF is a positive (negative) regulator.

### Construction of hierarchical regulatory network

We first built a core-hierarchy comprising of only the TFs using a breadth-first search algorithm in a bottom-up fashion in the following way. First, the TFs that were not regulated by any other TF were placed in the top layer. Next, the regulators that were regulated by the top TFs and also regulated other TFs were assigned to the middle layer. Finally, the regulators that did not regulate other TFs formed the bottom layer resulting in 3 layers of TFs. The interactions involving the miRNA were then added to these three layers. The miRNAs regulating the top TFs were placed in the top miRNA layer above the top layer TFs. Note that some of these miRNAs were regulated by lower layer TFs. Of the remaining miRNAs, the ones regulating the middle layer TFs were placed in the middle layer (between the top and middle layer TFs). From the set of remaining miRNAs, the ones that regulate the bottom layer TFs were placed in the lower layer (between the middle and bottom layer TFs). Finally, the remaining miRNAs were placed in the lowest layer; these did not regulate any regulators and only had incoming regulatory edges.

### Calculation of tissue specificity and stage specificity

Expression levels of all *C. elegans* genes at 8 different tissues at L2 stage were measured using tiling arrays [42]. The 8 tissues include poA, bone wall muscle, intestine, glr, GABA neurons, excretory cell, coelomocytes and panneural. The tissue specificity score (TSPS) for a gene is defined as 
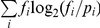
, where 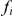 is the ratio of the gene expression level in tissue *i* to its sum total expression level across all tissues, and 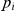 = 1/8 for all tissues, is the fractional expression of a gene under a null model assuming uniform expression across tissues. A greater tissue specificity score suggests more specific expression in a single or multiple tissues, whereas a score of zero suggests uniform expression. Apart from tissue specificity, the stage specificity score of a gene throughout its developmental time course is defined in a similar fashion.

## Supporting Information

Figure S1 Aggregation plots of binding signals for 22 worm transcription factors around the TSS of protein-coding genes (blue) and miRNA genes (green). The average binding signal of each TF across all coding genes and miRNA genes are shown. The curves for miRNA are more fluctuated due to the small number of miRNAs.
(PDF)

Figure S2 Overlapping of target coding genes among distinct transcription factors. The upper-left shows the number of shared target protein-coding genes between any pair of TFs. The lower heatmap shows the significance (−log10(P-value)) of overlapping based on hyper-geometric test.
(PDF)

Figure S3 Overlapping of target miRNAs among distinct transcription factors. The upper-left shows the number of shared target miRNAs between any pair of TFs. The lower heatmap shows the significance (−log10(P-value)) of overlapping based on hyper-geometric test.
(PDF)

Figure S4 Overlapping of target genes between transcription factors and miRNAs.
(PDF)

Figure S5 A list of sub-networks with 3 nodes in the integrated unsigned regulatory network. Only those sub-networks with at least one TF plus a miRNA or a protein-coding non-TF gene are shown.
(PDF)

Figure S6 A list of sub-networks with 3 nodes in the integrated signed regulatory network. Only those sub-networks with at least one TF plus a miRNA or a protein-coding non-TF gene are shown. The sign of a TF (positive/negative regulator) was inferred based on the correlation of its binding signal and the expression levels of down-stream genes.
(PDF)

Table S1 Properties of C. elegans transcription factors. Essentiality of transcription factors was determined by RNAi experiments. Tissue specificity and stage specificity were calculated based on the expression profiles of genes in 8 tissues at L2 stage and the developmental stage time course, respectively.
(XLS)

Table S2 A list of TF⇔miRNA feedback loops in C. elegans.
(XLS)

Table S3 A list of miRNAs located in a host-gene. The data was compiled based on miRBase database.
(XLS)

Table S4 Topological features and network motif analysis results for 8 integrated regulatory networks.
(PDF)
