# Supplementary figures and images for: Construction and Analysis of an Integrated Regulatory Network Derived from High-Throughput Sequencing Data
Source: PLoS Comput Biol. 2011 Nov 17;7(11):e1002190. doi: 10.1371/journal.pcbi.1002190 (PMC3219617; doi:10.1371/journal.pcbi.1002190)

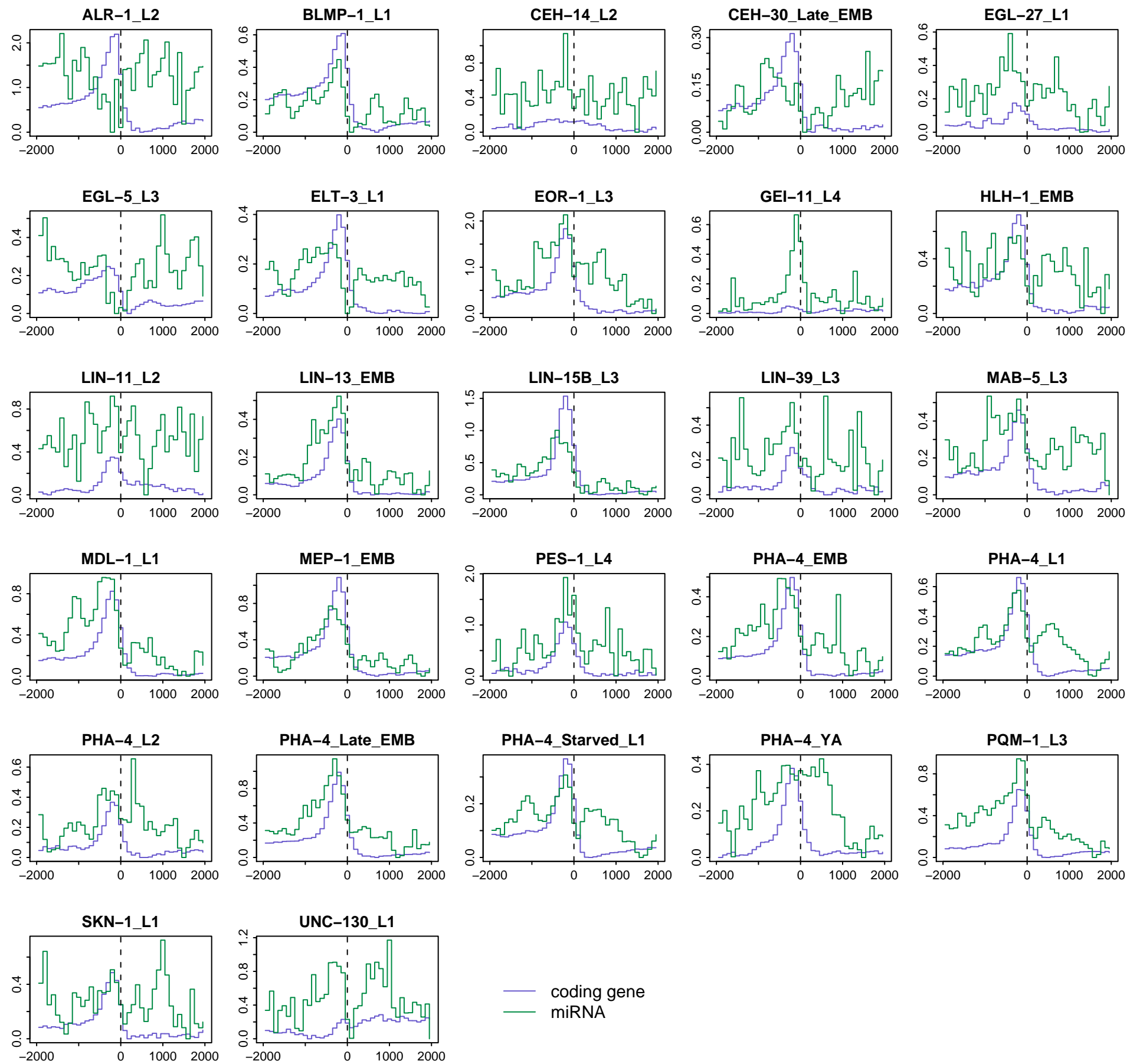

Supplement: Figure S1 — Aggregation plots of binding signals for 22 worm transcription factors around the TSS of protein-coding genes (blue) and miRNA genes (green). The average binding signal of each TF across all coding genes and miRNA genes are shown. The curves for miRNA are more fluctuated due to the small number of miRNAs. (PDF) [file pcbi.1002190.s001.pdf]

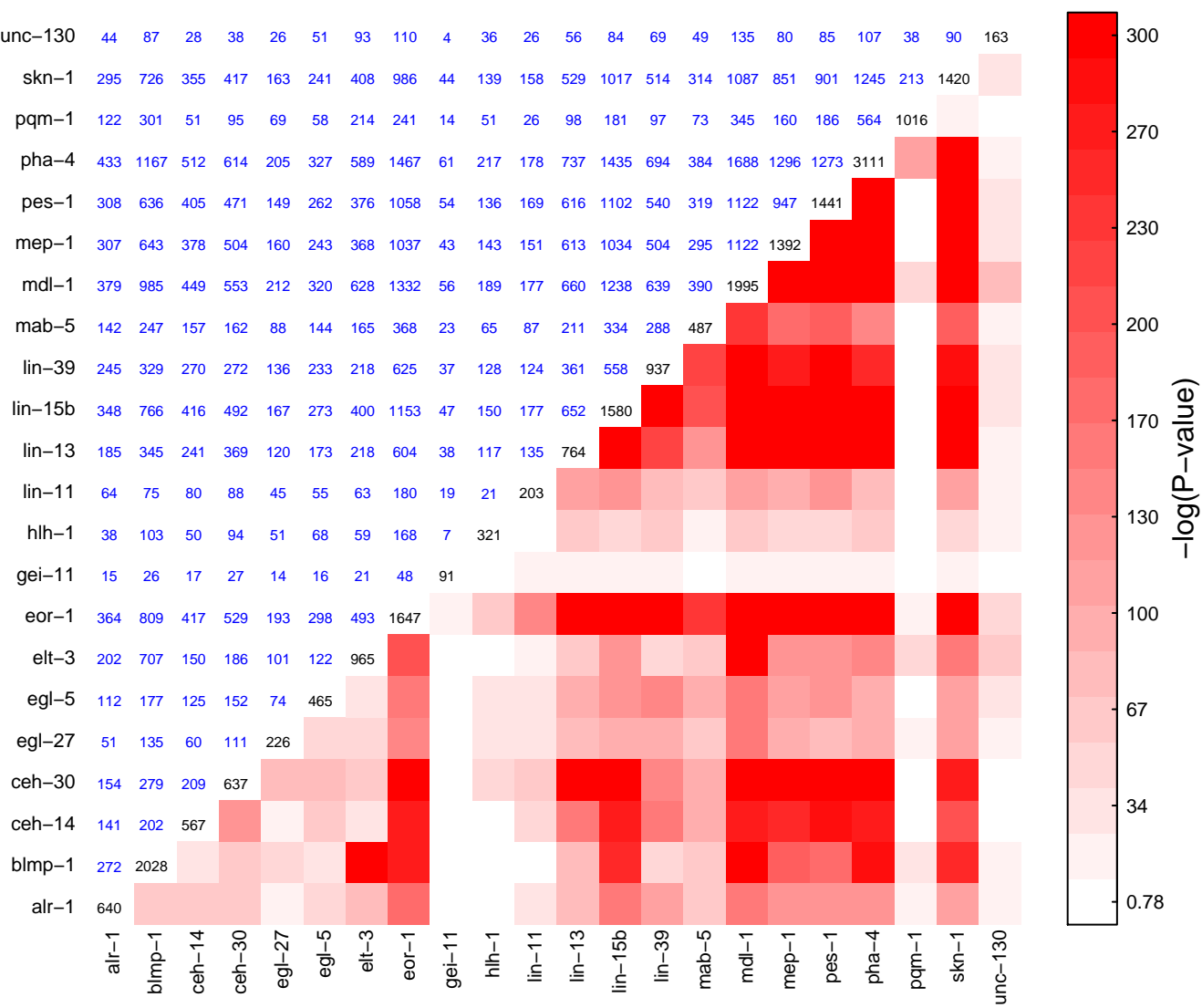

Supplement: Figure S2 — Overlapping of target coding genes among distinct transcription factors. The upper-left shows the number of shared target protein-coding genes between any pair of TFs. The lower heatmap shows the significance (−log10(P-value)) of overlapping based on hyper-geometric test. (PDF) [file pcbi.1002190.s002.pdf]

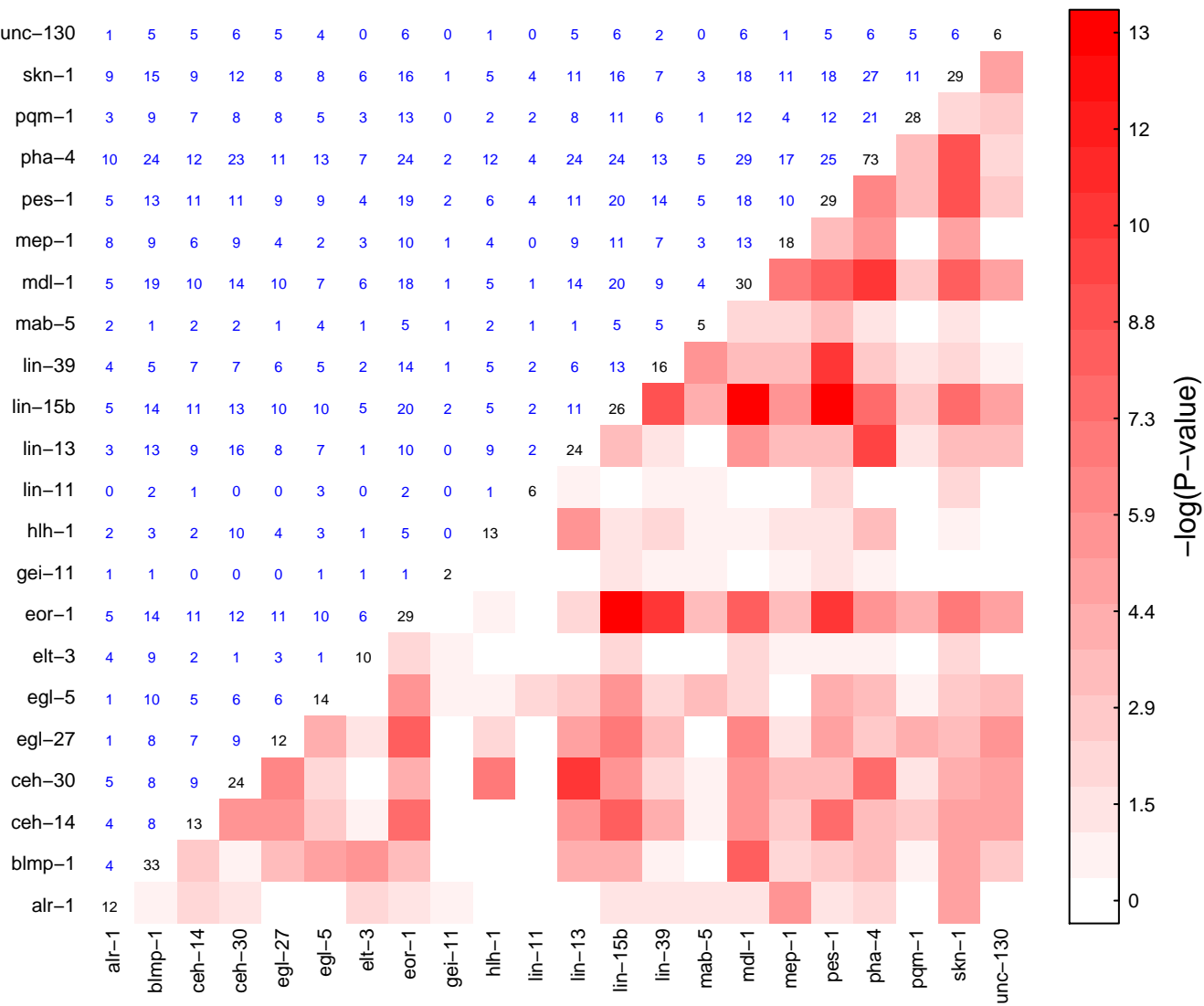

Supplement: Figure S3 — Overlapping of target miRNAs among distinct transcription factors. The upper-left shows the number of shared target miRNAs between any pair of TFs. The lower heatmap shows the significance (−log10(P-value)) of overlapping based on hyper-geometric test. (PDF) [file pcbi.1002190.s003.pdf]

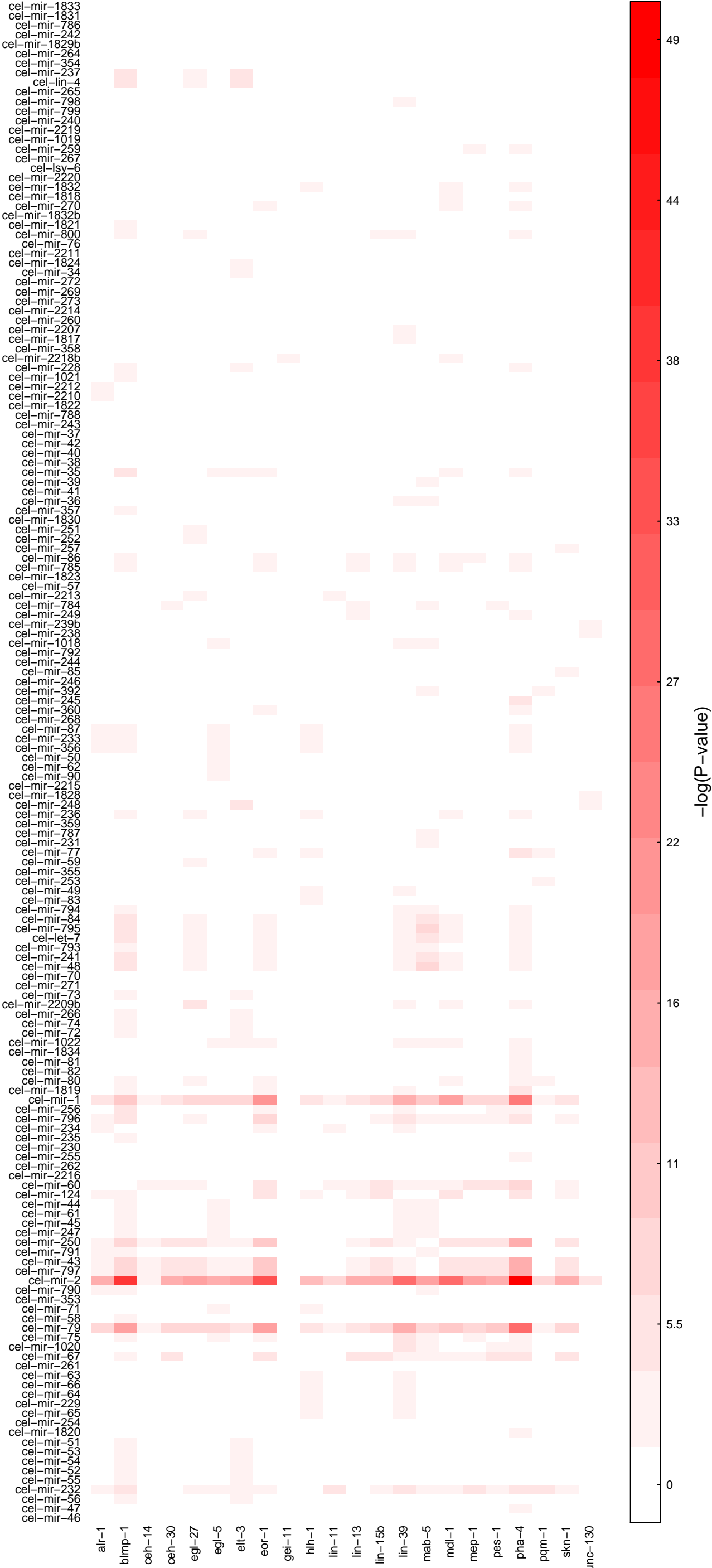

Supplement: Figure S4 — Overlapping of target genes between transcription factors and miRNAs. (PDF) [file pcbi.1002190.s004.pdf]

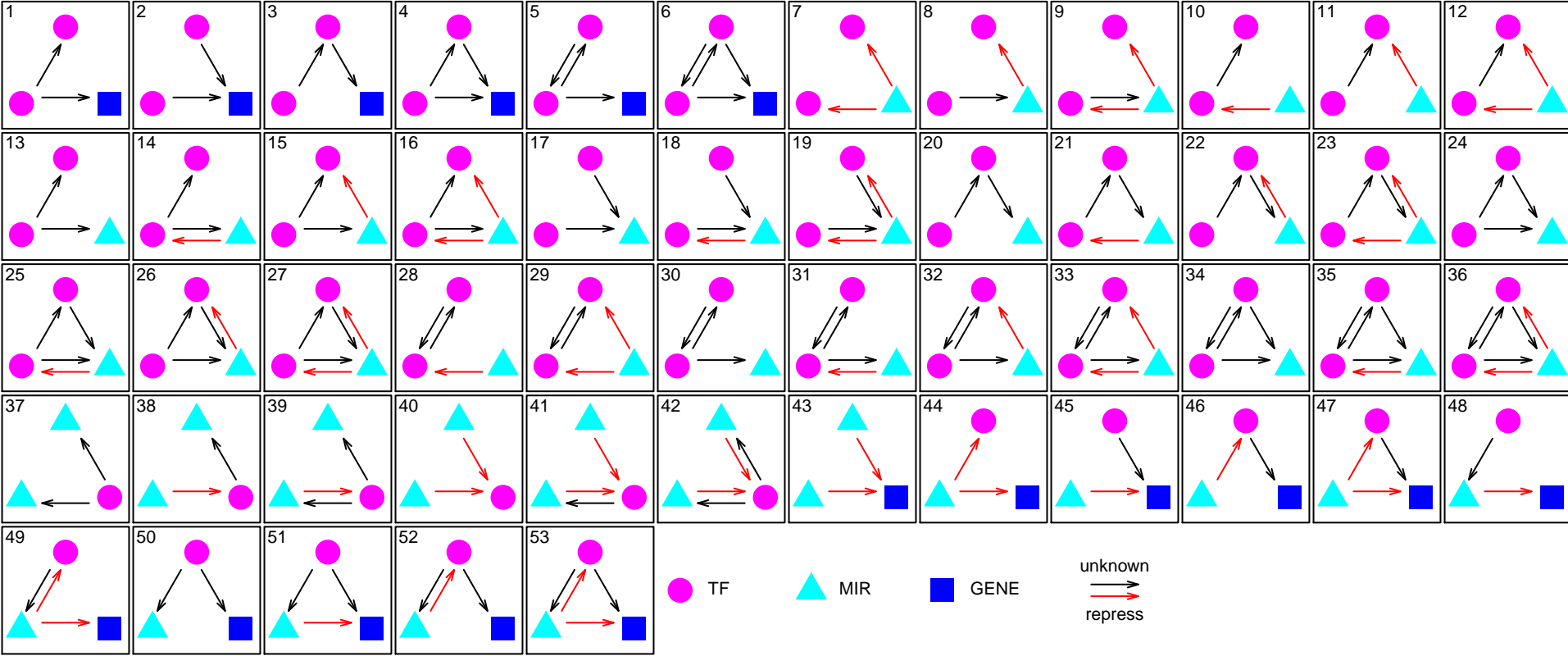

Supplement: Figure S5 — A list of sub-networks with 3 nodes in the integrated unsigned regulatory network. Only those sub-networks with at least one TF plus a miRNA or a protein-coding non-TF gene are shown. (PDF) [file pcbi.1002190.s005.pdf]

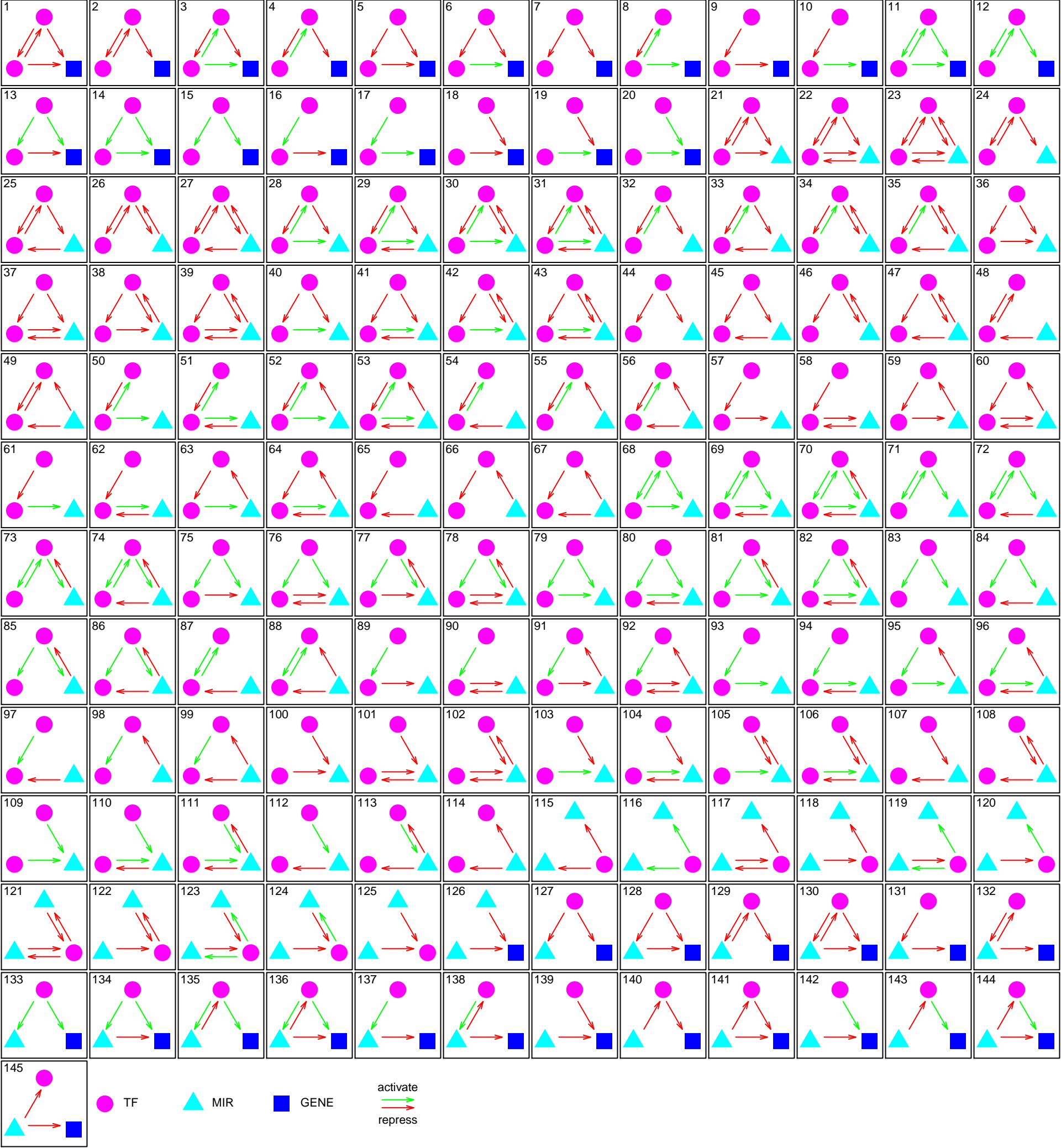

Supplement: Figure S6 — A list of sub-networks with 3 nodes in the integrated signed regulatory network. Only those sub-networks with at least one TF plus a miRNA or a protein-coding non-TF gene are shown. The sign of a TF (positive/negative regulator) was inferred based on the correlation of its binding signal and the expression levels of down-stream genes. (PDF) [file pcbi.1002190.s006.pdf]
