# Supplementary material for: Construction and Analysis of an Integrated Regulatory Network Derived from High-Throughput Sequencing Data
Source: PLoS Comput Biol. 2011 Nov 17;7(11):e1002190. doi: 10.1371/journal.pcbi.1002190 (PMC3219617; doi:10.1371/journal.pcbi.1002190)
Supplement: Table S4 — Topological features and network motif analysis results for 8 integrated regulatory networks. (PDF) [file pcbi.1002190.s010.pdf]

Note: case 7 is the one shown in the paper.

		**case1**	**case2**	**case3**	**case4**	**case5**	**case6**	**case7**	**case8**
**Parameter**	**Promoter**	[-500,300]	[-500,300]	[-1000,500]	[-1000,500]	[-500,300]	[-500,300]	[-1000,500]	[-1000,500]
	**HOT excluded**	No	No	No	No	Yes	Yes	Yes	Yes
	**miR target prediction**	3-way	5-way	3-way	5-way	3-way	5-way	3-way	5-way
**Topology**	**#TF**	376	342	413	384	360	320	393	357
	**#miR**	160	157	160	157	160	157	160	158
	**#Gene**	5295	4857	6059	5720	4874	4366	5574	5158
	**TF->TF**	1513	1513	1969	1969	1225	1225	1498	1498
	**TF->Gene**	21119	21119	27254	27254	16990	16990	20592	20592
	**TF->miR**	369	369	373	373	282	282	452	452
	**miR->Gene**	9011	2853	9011	2853	9011	2853	9011	2853

**P-value**		**case1**	**case2**	**case3**	**case4**	**case5**	**case6**	**case7**	**case8**
	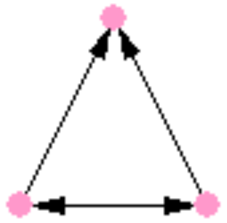	0.017	0.008	<0.001	<0.001	<0.001	<0.001	<0.001	<0.001
	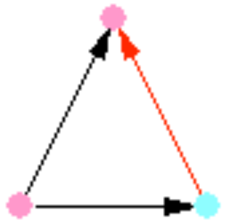	0.754	0.841	0.971	0.964	0.799	0.9	0.332	0.888
	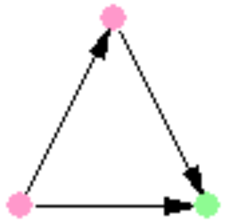	0.054	0.065	0.042	0.049	0.001	0.002	0.015	0.02
	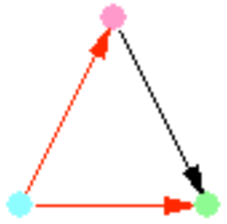	0.047	0.091	0.026	0.081	0.097	0.081	0.035	0.029

	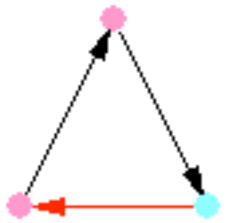	0.772	0.648	0.582	0.624	0.922	0.622	0.556	0.278
	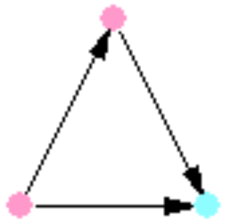	0.119	0.141	0.054	0.045	0.219	0.37	0.04	0.037
	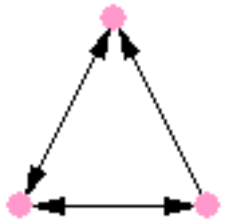	0.584	0.578	0.284	0.259	<0.001	<0.001	<0.001	<0.001
	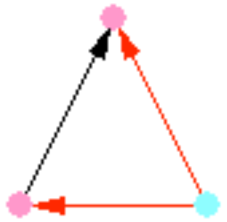	0.256	0.717	0.075	0.282	0.169	0.288	0.034	0.039
	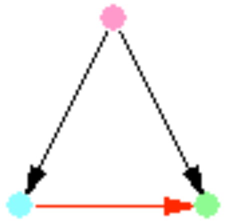	0.148	0.01	0.997	0.997	0.201	0.072	0.39	0.637
	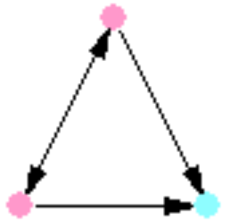	0.027	0.034	<0.001	<0.001	0.054	0.077	0.005	0.004
